# Ontological visualization of protein-protein interactions

**DOI:** 10.1186/1471-2105-6-29

**Published:** 2005-02-11

**Authors:** Harold J Drabkin, Christopher Hollenbeck, David P Hill, Judith A Blake

**Affiliations:** 1 Mouse Genome Informatics, The Jackson Laboratory, Bar Harbor, ME, USA; 2 Department of Computer Science, Rensselaer Polytechnic Institute, Troy, NY, USA

## Abstract

**Background:**

Cellular processes require the interaction of many proteins across several cellular compartments. Determining the collective network of such interactions is an important aspect of understanding the role and regulation of individual proteins. The Gene Ontology (GO) is used by model organism databases and other bioinformatics resources to provide functional annotation of proteins. The annotation process provides a mechanism to document the binding of one protein with another. We have constructed protein interaction networks for mouse proteins utilizing the information encoded in the GO annotations. The work reported here presents a methodology for integrating and visualizing information on protein-protein interactions.

**Results:**

GO annotation at Mouse Genome Informatics (MGI) captures 1318 curated, documented interactions. These include 129 binary interactions and 125 interaction involving three or more gene products. Three networks involve over 30 partners, the largest involving 109 proteins. Several tools are available at MGI to visualize and analyze these data.

**Conclusions:**

Curators at the MGI database annotate protein-protein interaction data from experimental reports from the literature. Integration of these data with the other types of data curated at MGI places protein binding data into the larger context of mouse biology and facilitates the generation of new biological hypotheses based on physical interactions among gene products.

## Background

### Protein networks

Cellular processes require the interaction of many proteins across several cellular compartments. Interactions can range in stability from persistent, such as between members of a stable complex, to transient, such as binding while being phosphorylated. Determining the collective network of such interactions should provide insight into which processes the individual members participate, and how they may be regulated.

Understanding protein interaction networks requires two steps. First, the interacting proteins must be identified, usually through some experimental methods. Secondly, the significance of the interaction networks needs to be assessed. Recently, there has been a focus on devising large scale screening methods to collect data on interacting proteins [1-3]. Additionally, several strategies have been used to predict networks based on small peptide interaction [4], analysis of co-evolution of protein families [5], analysis of orthology [6], and co-inheritance [7]. However, many of these types of studies are hindered by their inability to place the significance of the interaction networks in the broader biological context.

In addition to the large screening efforts, a significant amount of specific protein-protein interaction data has been reported in the literature over the years. Quite often, these studies report on only a few interacting proteins. It is difficult to place these isolated, yet specific reports in the larger biological context and interconnect them with other data. Recently, there have been efforts to extract such literature-based interaction information using text mining [8], or combinations of text mining and other predictive methods [9]. These then can be integrated into larger protein-protein interaction datasets. The work reported here presents a methodology for integrating and exploring information on protein-protein interactions.

### Model organism databases

Model Organism Databases (MODs) have been collecting diverse types of data about the genes and proteins from their respective organisms since the early 1990s (*e.g*. [10-13]). The goal of these databases is to integrate information about these organisms, placing experimental data in the context of the biology of the organism as a whole. Biological information on gene sequence, function, tissue-specific and developmental expression, as well as associated genetic and mutant phenotype data is incorporated into these systems. The documentation of protein-protein interactions and the integration with other data types allows potential for determining the significance of the interactions and placing these molecular interactions into greater biological context.

The Mouse Genome Informatics system (MGI) is the MOD for the laboratory mouse [14]. MGI integrates not only data used for GO annotation, but also data on a variety of aspects of mouse biology including gene sequence, orthologs, embryonic gene expression, alleles and their phenotypes, strains, and chromosome feature maps [15,16]. MGI provides highly curated information to the research community and to other bioinformatics resources [17].

### GO annotation

The Gene Ontology Consortium provides the biological community a structured vocabulary with which to enable consistent functional annotation of genes and gene products. [18]. Guidelines for the use of the GO vocabulary are provided by the Consortium [19]. Users of the GO are required to submit their annotations in a specified format, which is then made available to the public via the GO database [20]. Each annotation row lists the object being annotated, the GO term that is being assigned, an evidence code specifying the type of evidence that was used to make the assignment, and a reference. The format of the annotation includes the use of "modifier" fields which can be used either to modify the use of the term, or the use of the evidence code. One important modifier field is the "with" field. This field can be used to specify an external database link and provides the ability to qualify or support a given evidence code with a specific gene, nucleic acid sequence, protein sequence, or allele.

In the course of over six years, curators at MGI have made 79690 annotations to 15231 gene products using 3742 GO terms (All database statistics used in this paper are from the MGI release as of 7/30/04). The curation policy focuses on experiments in which the *murine *protein gene product is investigated. Many of the detailed annotations have been added on a paper-by-paper basis using the MGI literature collection that contains primary experimental information about mouse genes from over 90,000 references. The accumulation and use of these papers in annotation has been, for the most part, undirected. However, the structure of the GO and the relationships among terms allow grouping of the gene products that share common annotations. Such strategies may reveal hitherto unsuspected relationships between these proteins.

### Annotation with "protein binding"

"Protein binding" (GO:0005515), as used by the GO in the Molecular Function ontology, is defined as "interacting selectively with any protein or protein complex" [21]. This term has 70 sub-terms. A gene product can be annotated to "protein binding" using the IPI (inferred from physical interaction) evidence code and the "with" or "inferred from" field when the protein that it binds to has been specifically identified. In the case of the IPI evidence code, the "with" field requires a protein identifier, such as a SwissProt/Trembl ID (now UniProt). MGI curators use this evidence code to curate experimental evidence that demonstrates protein interactions

**Figure 1:**
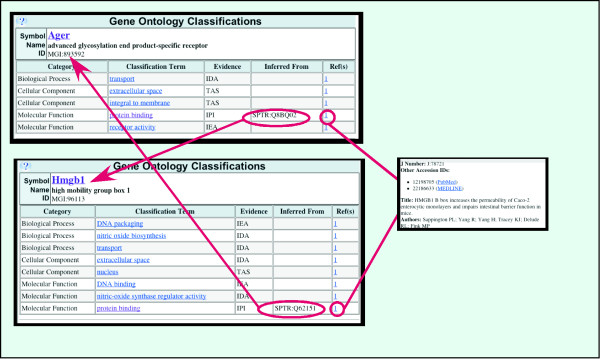
GO annotations for At and Lnk as displayed at MGI. The annotatons to GO:0005515, protein binding, are marked. The circled SPTR_ID points to the MGI marker that it is associated with. The two annotations share the same reference.

An example of GO annotation that includes "protein-binding" is shown for the gene product of *Ager*. In the case of *Ager *(advanced glycosylation end product-specific receptor, Figure 1), Takaki *et al*. [22] have demonstrated that the murine AGER protein binds to SPTR:Q8BQ02, the protein encoded by *Hmgb1 *(high mobility group box 1). A curator at MGI has captured this information in an MGI GO annotation for *Ager*. For completeness, a curator also annotated the gene product of *Hmgb1 *with "protein binding" with an IPI to SPTR:Q62151, the protein product of *Ager*, using the same reference. In this case, these are the only "protein binding" annotations for either of these proteins. These annotations represent an experimentally tested interaction of two proteins.

**Figure 2:**
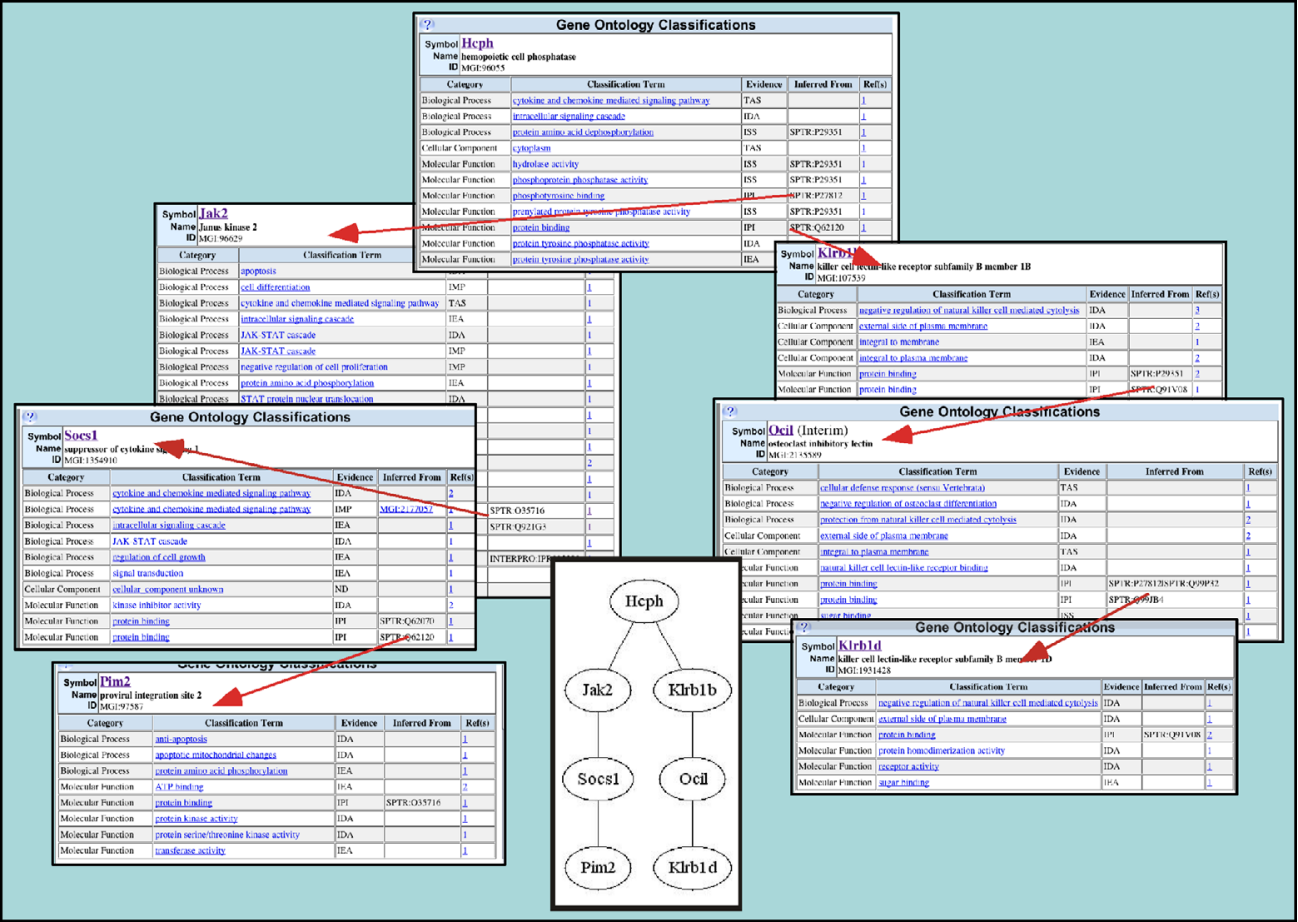
Construction of a simple protein interaction network using GO annotations to protein binding.

Beyond this specific reference, either of these two proteins could have further annotations from separate experiments reported in other references reporting binding to other proteins, which in turn have been annotated to binding to still others, thereby outlining a network of protein interactions. An example of a simple network is shown in Figure 2. The protein product of *Hcph *(hemopoietic cell phosphatase), has been shown to bind both the protein product of *Jak2 *(Janus kinase 2) ([23]) and Klrb1b (killer cell lectin-like receptor subfamily B member 1B) ([24]). JAK2 not only binds HCPH ([23]), but also SOCS1 (suppressor of cytokine signaling 1) [25], which in turn has been shown to bind PIM2 (proviral integration site 2) ([26]). KLRB1B has been demonstrated to bind OCIL (osteoclast inhibitory lectin) ([24]), which binds KLRB1D (killer cell lectin-like receptor Subfamily B member 1D) [27,24]. Thus, a seven member "network" has been described by integrating the data several independent investigations.

MGI has presently 1851 genes annotated to the term GO:0005515, "protein binding", or its sub-terms. These genes have 2247 annotations to this term, indicating that some of the gene products must bind more than one protein. These annotations were made independently over the years as curators entered data reference by reference. By collecting all of these annotation pairs, and identifying shared partners, it is possible to search for the presence of more complex networks that were not necessarily identified in each original piece of research literature.

## Results & discussion

### Discovery by inference

**Figure 3:**
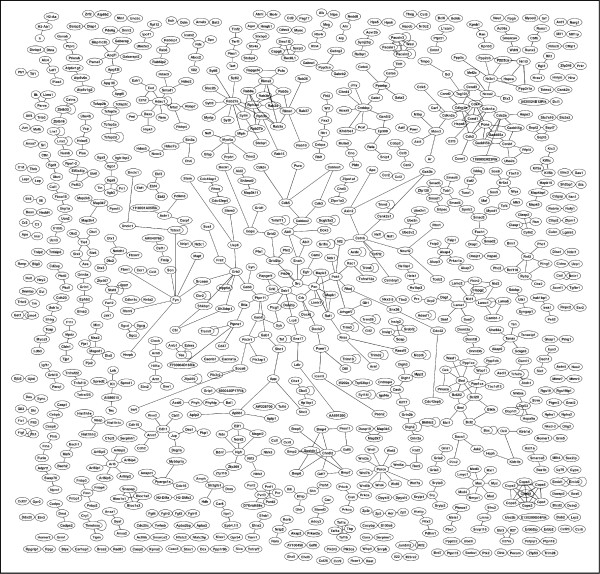
Murine protein-protein interaction catalog as documented by GO annotation to GO:0005515 protein binding.

**Figure 4:**
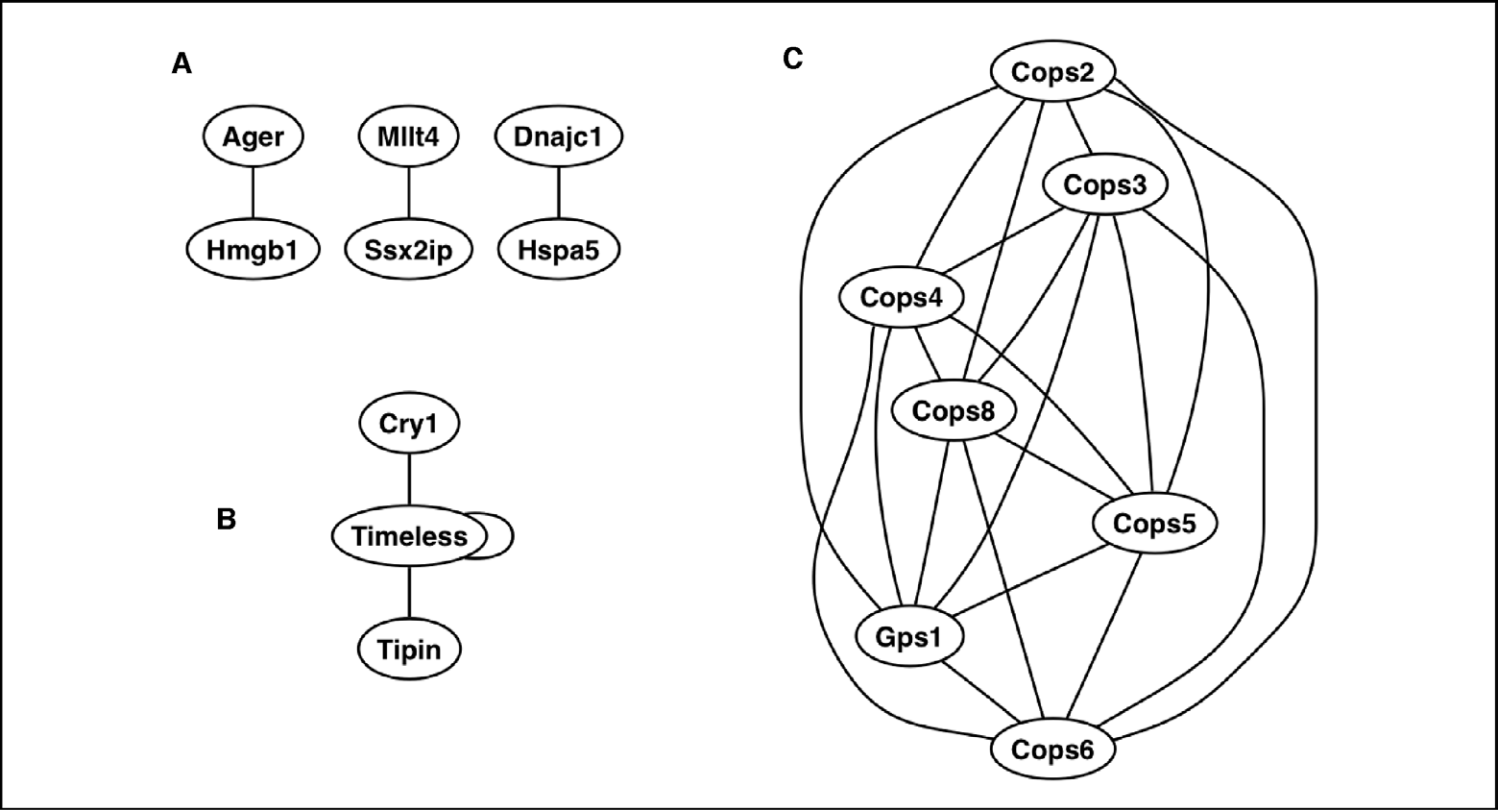
Selected interaction maps from Figure 3.

Figure 3 shows all 1318 annotated interactions captured by GO annotation. These include 129 binary interactions, and 125 interaction sets of three or greater. Figure 4 displays some of the associations in more detail. Figure 4A displays three sets of heterodimers. Figure 4B shows interactions among three proteins. Note the loop-back in the case of TIMELESS. This indicates that the protein forms a homodimer. Many of the annotation networks depict interactions among the subunits of protein and or riboprotein complexes. For example, Figure 4C shows the interactions of Cops (constitutive photomorphogenic) proteins homologs. These have been shown to assemble into a "signalosome complex" (GO:0008180) [28]. Thus, the GO data implicitly reveals connections among the many separate annotations to "protein-binding" made over the course of collecting data at MGI.

### Utilization of the interaction web to infer biological process information for experimentally uncharacterized genes (guilt by association)

There are instances in the annotations where a protein product has been shown to be able to bind another protein, but otherwise, nothing is known about the biological role of the protein. In these cases, MGI curators make an annotation to "protein binding", but also use a special annotation to indicate that nothing is known about the cellular location (GO:0008372, "cellular_component unknown") of the gene product or the process it is involved in (GO:0000004, "biological_process unknown"). A simple example is seen in the case of TIPIN (timeless interacting protein) (Figure 3B). It has been shown to bind the protein product of *Timeless*, a homolog of the *Drosophila *gene [29]. However, GO annotation of *Timeless *indicates that it is involved in biological processes of lung development and branching morphogenesis [30], and thus we would predict that *Tipin*, which is currently annotated to "biological_process unknown" might also play a role in these processes. Additionally, the Gene Expression index in MGI indicates that the *Tipin *is expressed in similar spatial and temporal patterns as *Timeless*, supporting the hypothesis that *Tipin *may be involved in similar processes. that the interaction may be significant [29]. These inferences can form the basis for directed experiments, such studying the effects of antisense RNA inhibition, as has been done for *Timeless *[30].

Cellular location may also be inferred from protein interactions. SOCS1 (suppressor of cytokine signaling 1) has "kinase inhibitor activity" (GO:0019210) and has been implemented in the "cytokine and chemokine mediated signaling pathway" (GO:0019221), and the JAK-STAT cascade (GO:0007259). However, its cellular location has not been documented in the available mouse literature. Analysis of the SOCS1 protein using predictive software such as Psort [31]) and SubLoc [32] predict that SOCS1 is a nuclear protein. However, there is as yet no direct evidence that this is so. The murine SOCS1 binds to JAK2 (Figure 3D[26]) which has been reported to be localized to the cytoplasm [33]. Therefore, we might expect that SOCS1 may also be localized to the cytoplasm. So, algorithmic evidence predicts that SOCS1 may also be localized to the nucleus and to the cytoplasm. These two independent predictions could stimulate investigations by direct experimentation. Although these types of analyses can be repeated for several proteins, their utility becomes unwieldy when analyzing networks larger than a few components.

### Analysis of larger interaction sets

**Figure 5:**
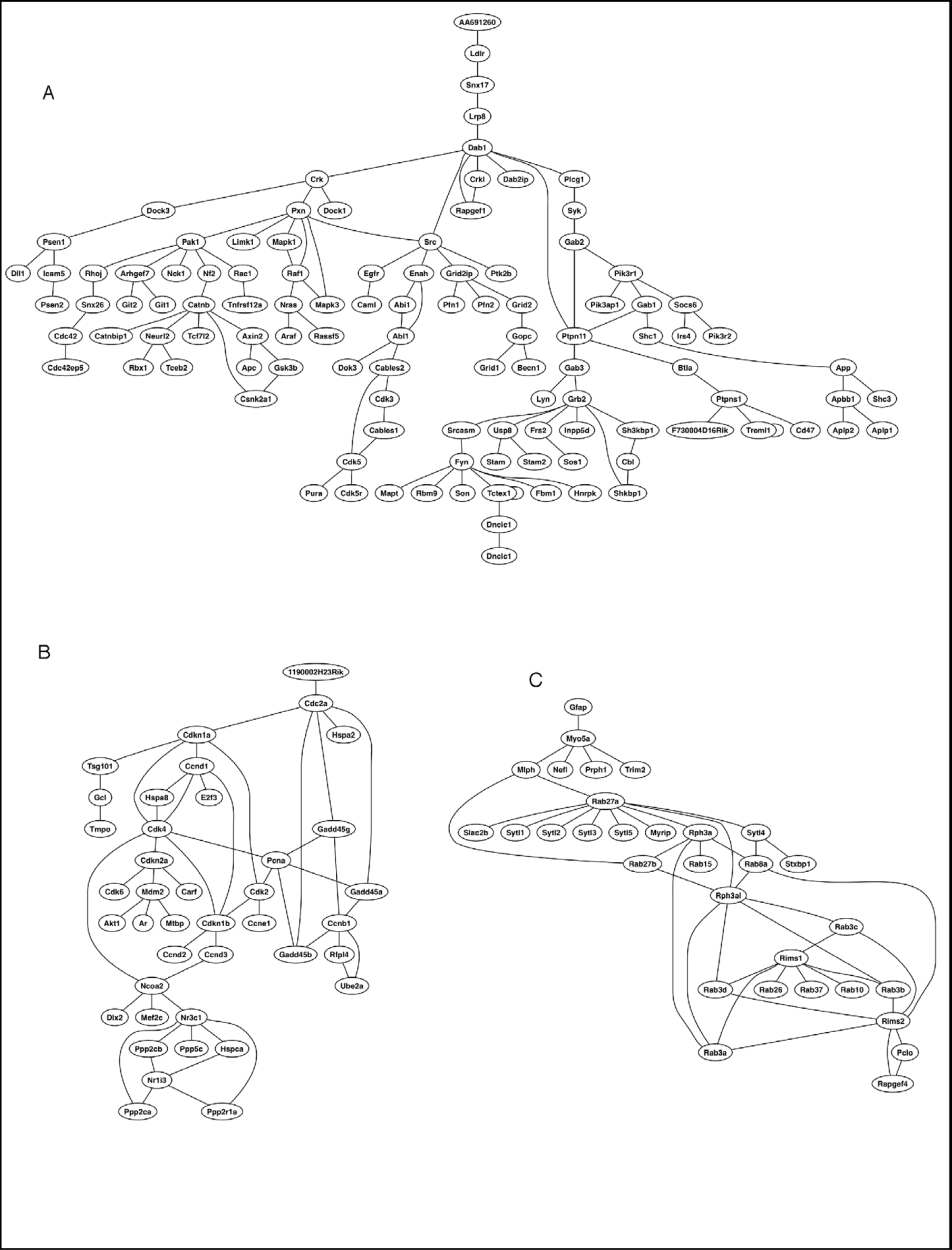
Interaction network maps showing 109 (A), 40 (B), and 31 (C) interacting proteins.

**Figure 6:**
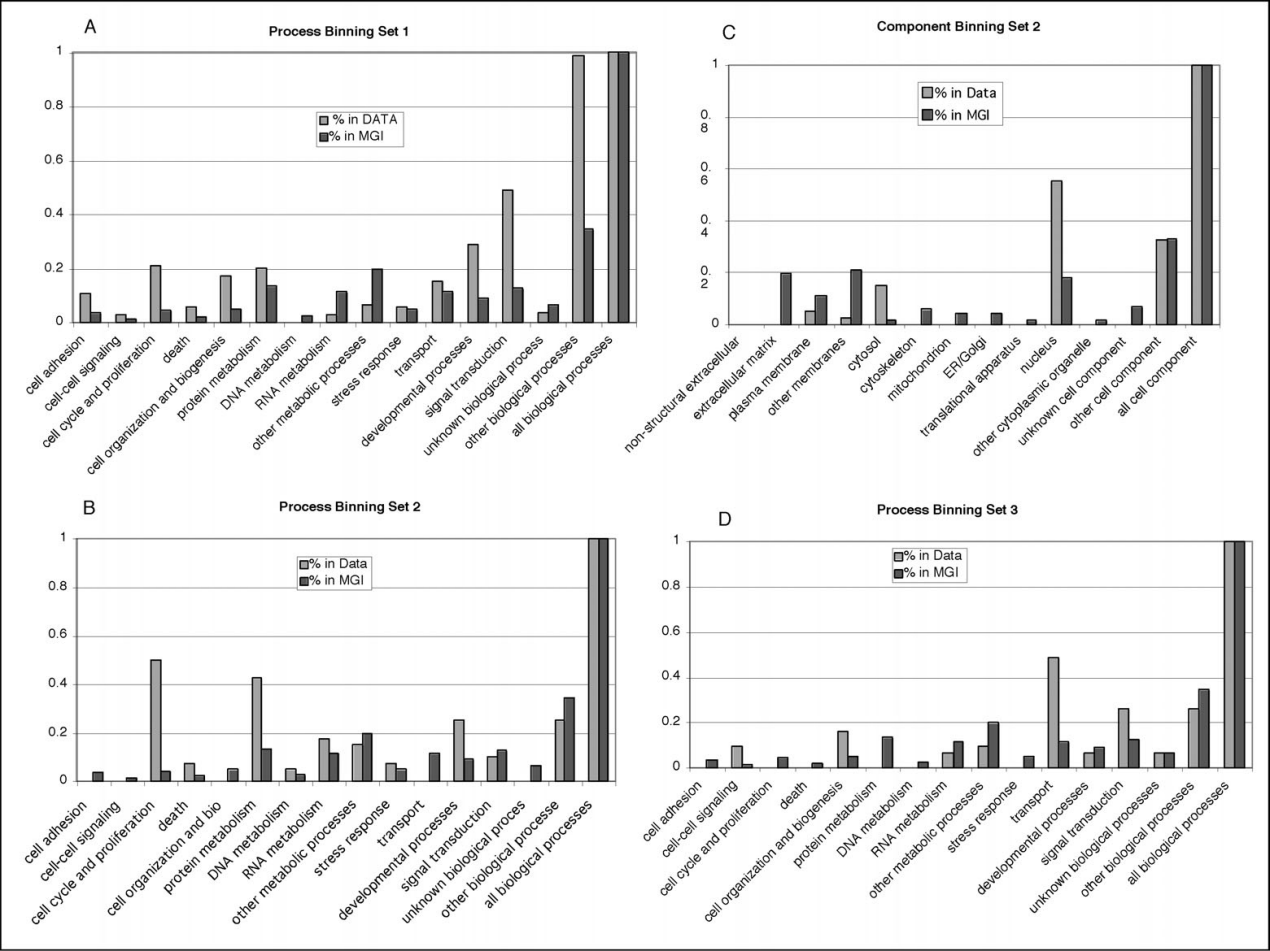
GO_Slim binning, displaying the faction of the total number of genes of either the data set or all genes in MGI falling into the indicated bins. Panel A, process binning for the 109 member set. Panels B and C, process and component for the 40 member set. Panel C, process binning for the 31 member set.

Three networks involve over 30 partners, the largest involving 109 proteins (Figure 5). Can we draw any inferences from these networks? Do they have anything in common? Several tools are available for using the GO in analysis and visualization of groupings of genes with respect to additional parameters after they have been selected by an experiment method, such as a microarray analysis, etc. In this case, our "method' is the mining of documented measurements of protein binding. These tools include GO_Term_Finder and GO_Slim Chart Tool) [34] Figure 6). The GO_Slim Chart Tool bins sets of genes based on shared annotations to specific predefined GO subtrees. It therefore reveals to a User the annotations that their genes have in common. The GO_Slim used for this study is summarized at the following site [35].

For the set of 109 proteins shown in figure 5A fifty-one of the gene products have annotations that fall into the "signal transduction" bin (Figure 6A). A number of the gene products in Figure 5B have been annotated to processes involved in proliferation (twenty proteins) and protein metabolism (seventeen), and twenty-two are nuclear (Figure 6B and 6C). Finally, fifteen of the gene products in the third largest set are involved in transport (Figure 6D). In all of these cases, one might begin to develop hypotheses to test whether the unannotated members of the networks may be involved in these processes.

Tools such as GO_Term_Finder [36] and its graphical counterpart Vlad [37] can be useful in finding commonality as well suggesting additional information about the roles of proteins in the cell which could be then tested experimentally. GO_Term finder computes the significance of the annotations for a selected set of genes within an annotation set compared to all the annotations of the entire set using a hypergeometric distribution algorithm. In this study, the entire set is the set of all genes in MGI with GO annotation. For example, for the 109 gene products shown in Figure 5A, thirty-two have process annotations for signal transduction or one of its subterms (p < 1.0E-23), suggesting that the interaction of the proteins may depict a large signal transduction network. Thirty-six of 109 gene products currently have either no annotation to the process ontology, or are annotated to "biological_process_unknown". These proteins may also be involved in the process of signal transduction. Seventeen the proteins depicted in the 40-member network (Figure 5B) have been annotated to "regulation of the cell cycle" (GO:0000074, p < 1.0E-26). Therefore 1190002H23Rik is likely involved in regulation of the cell cycle. Further support for this is that this protein has been annotated to be involved in the "cell cycle" based on sequence similarity to human RGC32 [38].

Finally, twelve of the proteins displayed in Figure 5C have annotations to exocytosis or its children in common (GO:0006887, p < 1.0E-23).

The networks suggested by the collection of annotations to this GO term involve interactions that are more or less stable under experimental conditions. A gene product is shown to have protein binding activity by a variety of direct assays such as yeast two-hybrid screening [39], co-immunoprecipitation and other immunoaffinity methods [40], GST-or other tag pull-down assays [41], fluorescence resonance transfer [42], or other direct measurements [43]. Due to the nature of some of the assays, caution must be taken when attributing significance. For example, false positives may obtained from yeast two-hybrid assays for a variety of reasons [44]. Therefore, confirmation by other methods, such as co-immunoprecipitation, may strengthen the likelihood of the implied interaction. Currently, the GO annotation does not allow for the capture of any distinction among these assays, with the result that they are all included together. Despite these serious considerations, large data sets can be effectively examined using these procedures and the results can provide a basis for directed hypotheses and experimentation.

### Integration with MGI

The Mouse Genome Informatics system integrates not only data used for GO annotation, but also data on a variety of aspects of mouse biology including embryonic gene expression, alleles and their phenotypes, and chromosome location. The integration of these datasets allows for complex queries, such as "list all genes expressed in the liver at Tyler Stage 15, located on chromosome 12, annotated to "protein binding" AND "nucleus". The integration of protein-protein network visualization into such queries can aide in determining the significance of more complex interaction networks. By combining the above query with our graphical tools, it is possible to get a graphical view of all protein interaction networks in the nucleus of a 9.5 dpc mouse embryo. As annotation progresses and becomes more complete, these types of queries will become more and more informative.

During the generation of the interaction sets, it was found that programs such as Graphviz, could easily visualize missing annotations based on the interaction of two proteins. When information about a protein comes from different sources, a curator that is curating a single reference may not necessarily record all of the information implied by a physical interaction, such as cellular location in the example above. Views such as Graphviz can help curators to spot missing data and they may at some point be useful in themselves to display annotations.

MGI curators aggressively adopted the use of the "with" field when annotating to "protein binding" during the early stages of annotation efforts at the database. Similar networks may also be mined from the GO data sets available from the other model organism databases participating in the GO. Recently, Lehner and Fraser used GO annotation to analyze a human interaction set predicted from orthology to yeast, *Drosophila*, and *C. elegans *interaction sets [45]. The GO is used by many species-specific organism databases to annotate gene products. The use of these annotation sets to construct species-specific interaction will compliment curated interaction resources such as BIND [46] and HPRD [47] to guide hypothesis generation in suggesting specific experimental investigations.

## Conclusions

We have demonstrated that functional annotations curated via GO hierarchies can be used to obtain a summary set from independent annotations to "protein-binding" to form protein-protein interaction networks. The members of these protein-protein interaction sets can be further examined for additional shared GO annotations. Integration of these data with the other types of data curated at MGI places protein binding data into the larger context of mouse biology and will aid in the discovery of new biological knowledge based on physical interactions among gene products.

## Methods

Gene annotations for protein binding interactions are made by manual inspection of published literature. In every case, experimental evidence is supplied in the manuscript to support the interaction that is reported. Annotation of genes to other GO terms is made by a variety of methods including the conservative translation of functional information contained in SwissProt protein records, conservative inference from InterPro domains, and manual curation of the published literature.

Data was obtained from the Mouse Genome Informatics system by use of custom SQL queries to collect all markers that had been annotated to "protein binding" or its children using the IPI evidence code. The protein sequence identifier in the "inferred from field" was matched to the appropriate gene in the database. The final output consisted of a two-column file with column 1 being the first protein, and column 2 the protein it binds. This formed the basic data set that was passed to Graphviz [48] for display. Additional Perl scripts were used to separate out each individual network.

The two column lists were also used as the basis for data files listing all unique genes in each network. These were then used for input files for GO_Slim Tool [34] and GO_Term finder [36]. These files are available on the MGI ftp site .

GraphViz on the Macintosh OS X platform is a product of Pixelglow [49]. GraphViz is an open source program made available by ATT [50].

## Authors' contributions

HJD conceived of and designed this study and implemented the graphical displays using Graphviz and analyzed the significance of the results. He has also been involved in contributing to the GO annotations. CH devised the Perl scripts used to parse out individual interaction sets and removal of redundancies. DPH helped to draft the manuscript and contributed to the annotations. JAB has been involved in the overall design of the GO project at MGI and of gene annotations in MGI overall, and has added critical revisions for important intellectual content to this paper.
